# A Strategy for When Two Rare Entities Strike at Once: Carotid Artery Dissection With Carotid Sinus Hypersensitivity and Syndrome

**DOI:** 10.7759/cureus.87813

**Published:** 2025-07-13

**Authors:** Hassan Ur Rehman, Adrienne A Jonathan, Sadia Haroon, Soha Mahran, Kamil Betkowski

**Affiliations:** 1 Internal Medicine, University Hospitals of Morecambe Bay NHS Foundation Trust, Lancaster, GBR; 2 Cardiology, University Hospital Galway, Galway, IRL; 3 Radiology, University Hospitals of Morecambe Bay NHS Foundation Trust, Lancaster, GBR

**Keywords:** cardiac arrhythmia, carotid sinus hypersensitivity, carotid sinus syndrome, ischemic cerebrovascular disease, permanent pacemaker implantation (ppm)

## Abstract

Carotid artery dissection involving the sinus region can lead to both neurologic and cardiovascular complications. We present the case of a previously healthy, middle-aged man with no known risk factors who arrived at the Emergency Department with an acute middle cerebral artery infarct. During hospitalisation, he developed recurrent syncope, bradycardia, and hypotension - unresponsive to carotid sinus massage. Imaging confirmed a carotid artery dissection extending into the carotid sinus. While stroke management was initiated, ongoing cardiovascular instability required the placement of a permanent pacemaker. This case emphasises the need to consider carotid dissection in stroke patients without typical comorbidities, and to recognise carotid sinus involvement as a cause of severe autonomic dysfunction.

## Introduction

Approximately 85% of ischaemic strokes are secondary to small or large vessel atherosclerosis and cardio-embolism, and those in younger patients may occur due to extracranial dissection; the remaining 15% of cases are categorised as intracerebral haemorrhages [1]. The incidence of ischaemic stroke secondary to dissection is currently increasing, but whether there is an association with atherosclerosis has yet to be determined [2]. Internal carotid artery (ICA) dissection is an important and underdiagnosed cause of stroke, particularly in younger individuals, accounting for up to 20% of ischaemic strokes in patients under 45 years of age [3]. Among these, right-sided ICA dissection resulting in artery-to-artery embolism and completed infarction is relatively rare. Most dissections tend to remain localised, presenting with symptoms such as ipsilateral headache, neck pain, or Horner's syndrome. However, in some cases, an intramural haematoma can serve as a source of thromboembolism, leading to distal vessel occlusion and cerebral infarction [4]. Clinical trials, including treatment for such strokes, have not concluded whether anticoagulants or antiplatelets are the superior choice of treatment. Nevertheless, cerebral perfusion should be the priority in such cases, to avoid cerebral insult and ischaemic stroke [5].

Carotid sinus hypersensitivity (CSH), due to carotid sinus compression at the bifurcation of the carotid arteries, results in significant hypotension, bradycardia, and even fatality. The spot where the internal and external carotid arteries meet is a dilated area containing baroreceptors. These receptors regulate heart rate (HR) and blood pressure (BP) via parasympathetic activation and other mechanisms. Carotid artery dissection may lead to CSH through mechanical distortion or stretching of the carotid sinus baroreceptors. Additionally, ischaemia of the afferent nerves innervating the sinus region (e.g., glossopharyngeal or vagus nerves) may enhance baroreflex sensitivity, predisposing individuals to reflex bradycardia and syncope [6,7].

Transcranial Doppler studies have demonstrated the presence of microembolic signals in a significant proportion of patients with extracranial ICA dissection, reinforcing the embolic mechanism in such cases [8].

This case presents a rare clinical overlap between carotid artery dissection and cardioinhibitory carotid sinus syndrome (CSS), resulting in both ischaemic stroke and recurrent symptomatic bradycardia. We aim to highlight this unusual but clinically significant association, emphasise the diagnostic and management challenges it presents, and contribute to the limited literature on the autonomic complications of carotid artery dissection.

While ICA dissection is a known cause of ischaemic stroke, cases involving right-sided ICA dissection leading to artery-to-artery embolism and subsequent stroke evolution and completion are uncommon. The majority of spontaneous dissections are either asymptomatic or present with localised symptoms, such as headache or neck pain. Embolic phenomena causing large vessel occlusion and progressive infarction, as seen in this case, represent a rare but clinically significant complication that warrants prompt recognition and targeted management.

Written informed consent for publication was obtained from the patient using the BMJ patient consent form, which conforms to internationally accepted ethical standards for the publication of case reports.

## Case presentation

On February 18, 2024, a 55-year-old White British male presented to the Emergency Department of the Royal Lancaster Infirmary with sudden-onset left-sided facial droop, left arm paresthesia, and uncoordinated gait, prompting immediate assessment and intervention. His symptoms began at around noon, when he noticed dysphasia, left-sided facial droop, and sensory disturbances in the left arm. His family history included a transient ischaemic attack. His past medical history included well-controlled asthma, with no regular medications. He was a non-smoker and drank one to two units of alcohol approximately four times per week.

**Figure 1:**
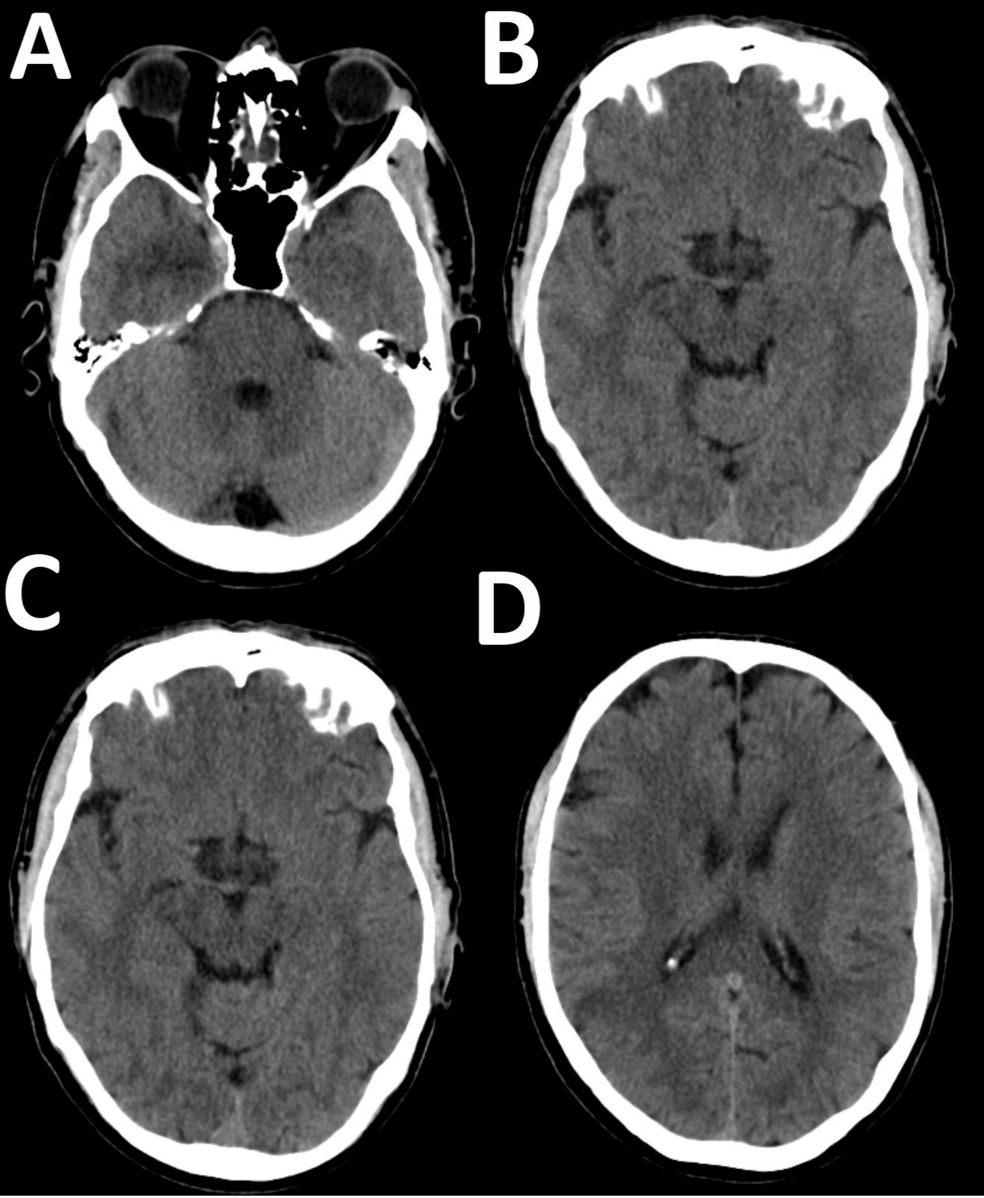
Computed tomography (CT) scan of the head showing no intracranial haemorrhage. (A) Posterior fossa; (B) midbrain; (C) thalamus and temporal lobes; (D) lateral ventricles and centrum semiovale

Physical examination revealed left inferior quadrantanopia, past pointing, and paresthesia in the left upper limb. Initial vital signs were within normal limits, with a National Early Warning Score (NEWS) of 0, respiratory rate (RR) of 18, oxygen saturation (SpO₂) of 97% on room air (RA), BP of 168/98 mmHg, HR of 86 bpm, and temperature of 37.5°C. An initial non-contrast computed tomography (CT) scan of the head showed no evidence of acute intracranial haemorrhage (Figure 1).

Initial management, as per the NICE (National Institute for Health and Care Excellence) (UK) stroke guidelines, included a stat dose of 300 mg of oral aspirin, followed by 300 mg once daily for the next two weeks; lansoprazole 15 mg once daily for three weeks; and oral atorvastatin 80 mg once daily, to be taken lifelong. Initial blood tests - including venous blood gas, complete blood count, C-reactive protein, and renal and liver function tests - were within normal ranges. 

**Figure 2:**
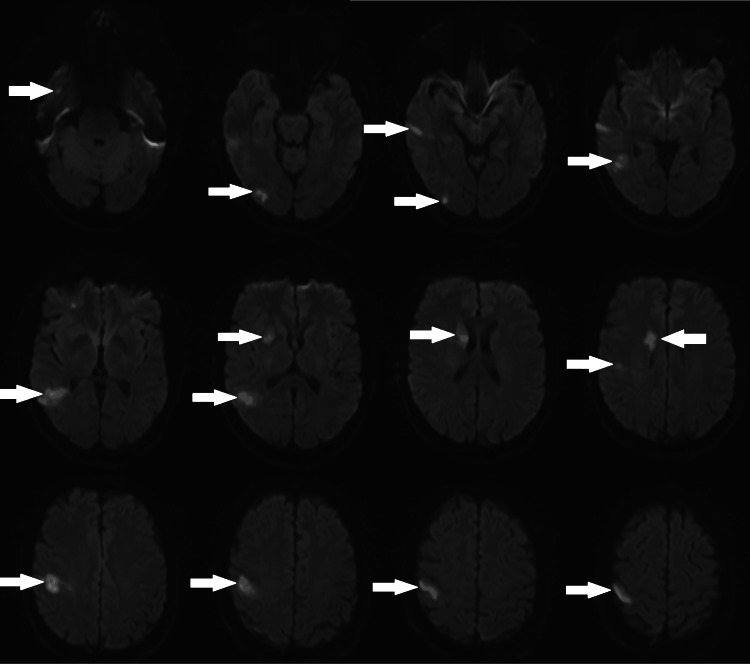
Axial MRI DWI baseline with arrows showing the initial right anterior circulation infarct. MRI DWI, Magnetic Resonance Imaging Diffusion-Weighted Imaging

The following day, magnetic resonance imaging (MRI) demonstrated restricted diffusion consistent with ischaemia in the right anterior temporal lobe (middle cerebral artery, or MCA), right posterior temporal lobe (posterior cerebral artery (PCA) territory; MCA/PCA watershed area), right inferior frontal lobe (MCA territory; anterior cerebral artery (ACA)/MCA watershed), right putamen and head of caudate nucleus (anterior choroidal artery), and right parietal lobe/postcentral gyrus (MCA), as seen in Figure 2.

**Figure 3:**
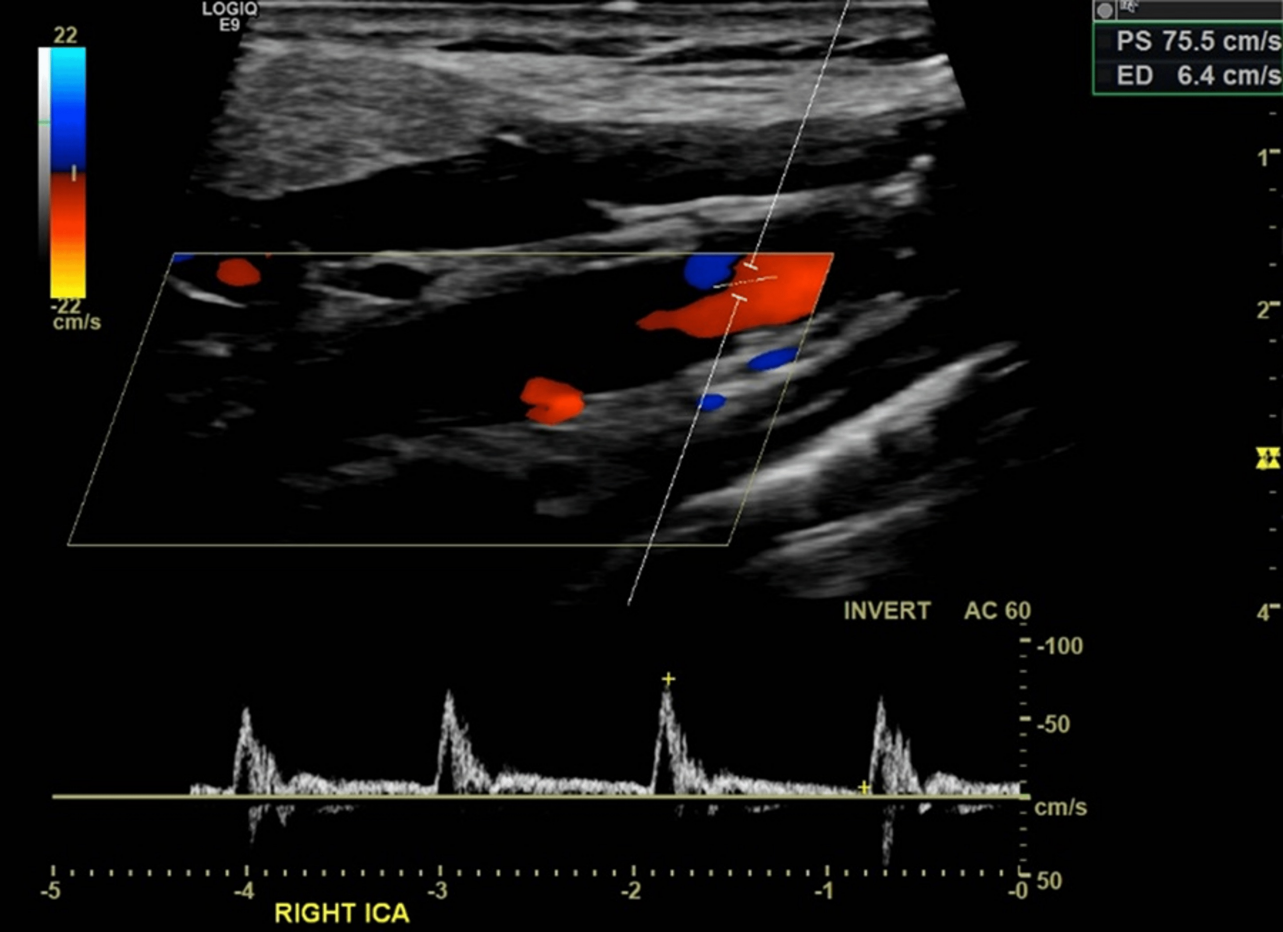
Doppler ultrasound of the right ICA demonstrated a lack of colour flow within the vessel, with a proximal 'flame sign,' along with bi-directional flow (red and blue signal within the vessel). ICA, Internal Carotid Artery

Inpatient spectral Doppler of the vessel proximal to the obstruction demonstrated slight spectral broadening (filling in of the systolic waveform indicating turbulent flow), a peak systolic velocity (PSV) of 75.5 cm/s (normal 60-100 cm/s), and an end-diastolic velocity (EDV) of 6.4 cm/s (normal <40 cm/s). No lower limit of EDV is agreed upon in the literature. However, in this case, the EDV was likely reduced, as the resistive index ($RI = \frac{PSV - EDV}{PSV}$) was elevated at 0.92 (normal range: 0.55-0.7), consistent with a distal obstruction. Mean velocity was not recorded for this examination, and it is not possible to calculate the pulsatility index ($PI = \frac{PSV - EDV}{\text{mean velocity}}$), as seen in Figure 3.

**Figure 4:**
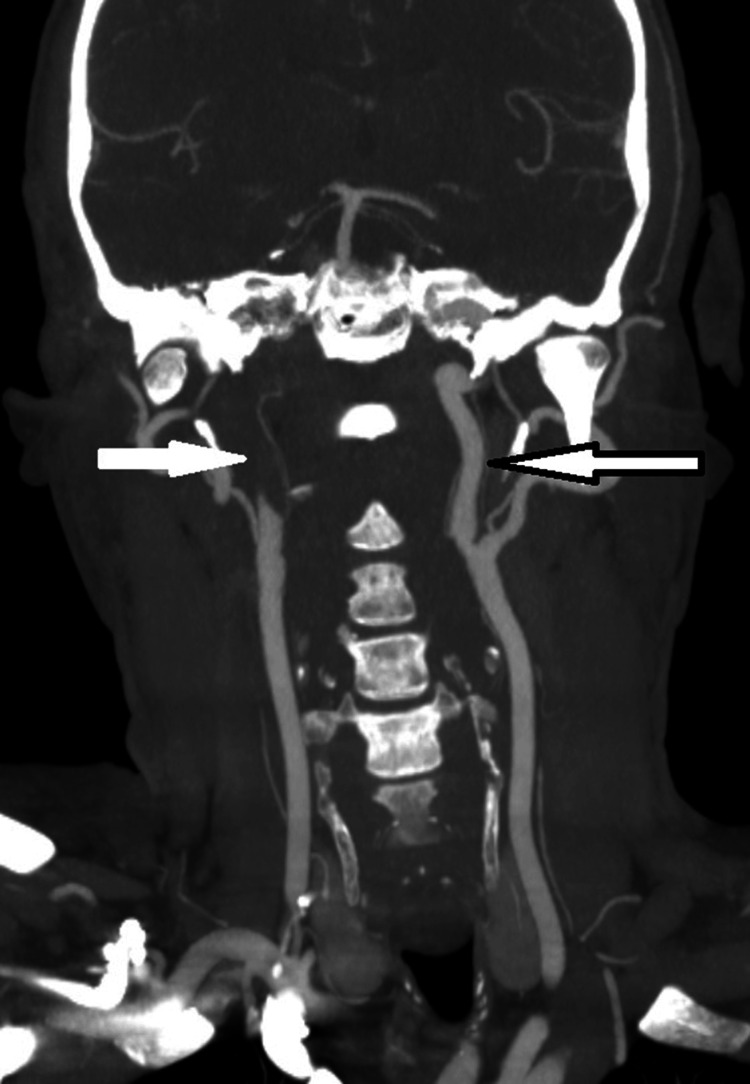
CT angiogram (coronal view) of the aortic arch and carotids, revealing complete occlusion of the right ICA (white arrow), and a patent left ICA (arrow with black borders), approximately 1.5 cm from the bifurcation. CT, Computed Tomography; ICA, Internal Carotid Artery

CT angiography demonstrated a filling defect in the right ICA, with a ‘flame sign’ tapering of the proximal ICA - a classic finding in ICA dissections. Other features that favoured dissection, as opposed to atherosclerotic thromboembolism, included a long-segment filling defect, no vessel calcification to suggest atherosclerotic disease, and vessel narrowing beginning beyond the vessel origin (as opposed to most atherosclerotic narrowings, which begin at the ostium). There was also a complete occlusion of the right ICA, raising suspicion for underlying vascular pathology, notably aortic dissection, as seen in Figure 4. 

Given the lack of atherosclerotic disease elsewhere, the ischaemic stroke was likely secondary to the dissection. The patient’s final diagnosis was multiple right-sided territory ischaemic infarcts, secondary to right carotid artery dissection. The patient was placed on telemetry, as per protocol, which revealed episodes of sinus bradycardia with HRs ranging from 47 to 69 bpm.

**Figure 5:**
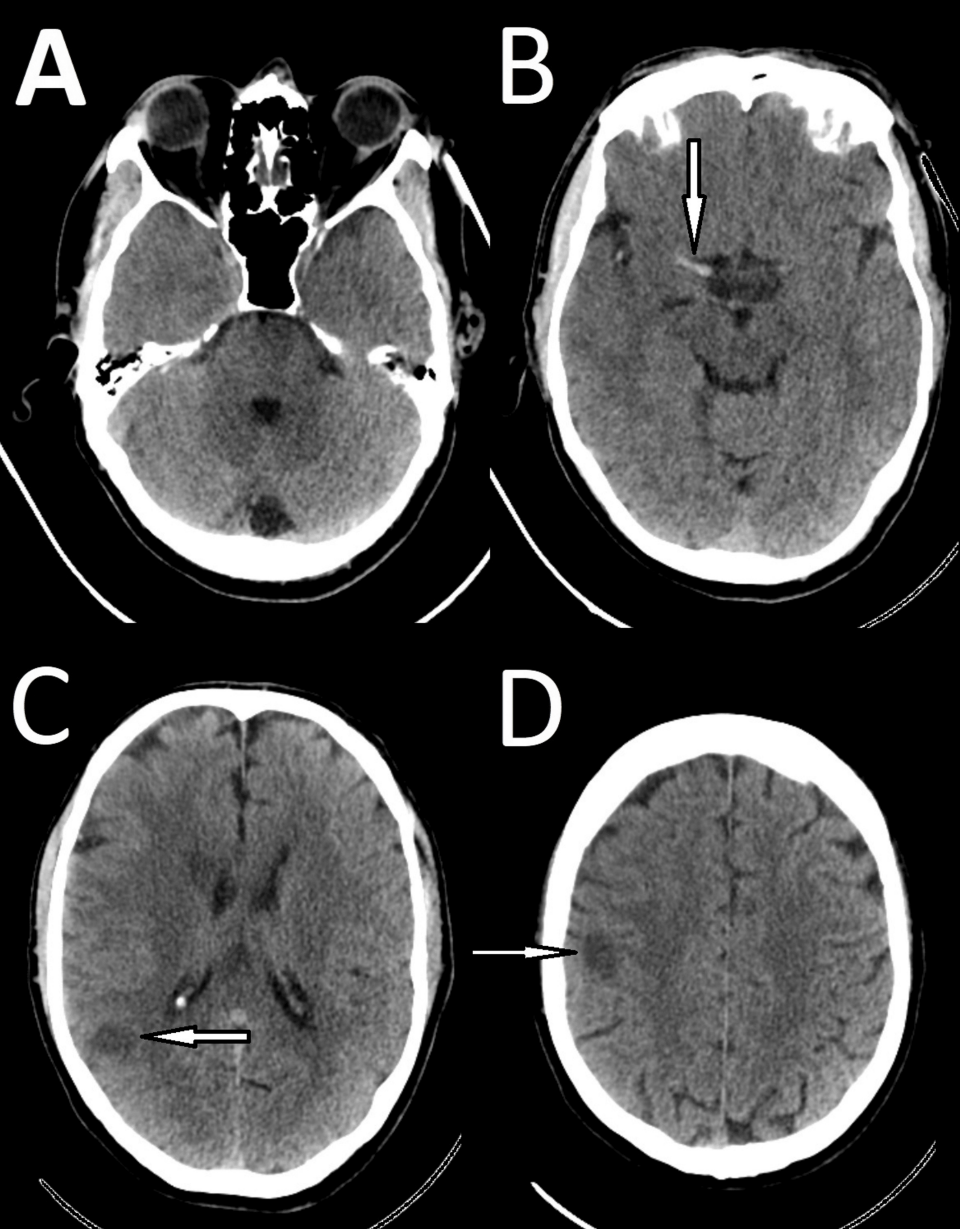
(A-B) Acute infarct (thrombus) in the head of the right caudate nucleus (medial lenticulostriate arteries, ACA); (C-D) arrows showing acute infarct (thrombus) in the right lentiform nucleus (lateral lenticulostriate arteries, MCA). ACA, Anterior Cerebral Artery; MCA, Middle Cerebral Artery

The following day, he had a sudden left upper limb and left-sided facial weakness. Examination revealed left-sided hemiplegia, with a power grading of 1 (out of 5) based on the Medical Research Council Scale for Muscle Strength. His NEWS remained at 0, with an RR of 18, SpO₂ of 98% on RA, BP of 155/102 mmHg, HR of 68 bpm, temperature of 36.5°C, and a Glasgow Coma Score of 14/15 (E3 V5 M6). At this stage, we suspected that the patient was experiencing a recurrent ischaemic event, likely due to artery-to-artery embolism secondary to thrombus formation and migration from the dissected segment of the ICA. The stroke was classified as TOAST subtype 4: stroke of other determined aetiology (non-atherosclerotic arteriopathy). A repeat CT brain scan showed an acute right MCA and ACA territory infarct, with considerably increased density compared to the previous scan (Figure 5).

According to our protocols, the patient was not scheduled for thrombolysis due to the previous MRI findings of multiple acute infarcts within the last 24 hours. The case was discussed with the neurosurgery team to evaluate the need for vascular repair. However, based on imaging findings and the patient’s clinical status, neurosurgery advised that there was no indication for immediate surgical management.

The next day, he began to have lengthy cardiac pauses on telemetry, continuing into the early hours of the morning. Within the space of one hour, he had pauses lasting from 11 to 22 seconds, during which it appeared he had lost consciousness for a few minutes. After discussing this with the cardiology team, an isoprenaline hydrochloride infusion at 8 mL per hour (2 mg/500 mL) was started. We decided to continue the infusion for 24 hours.

**Figure 6:**
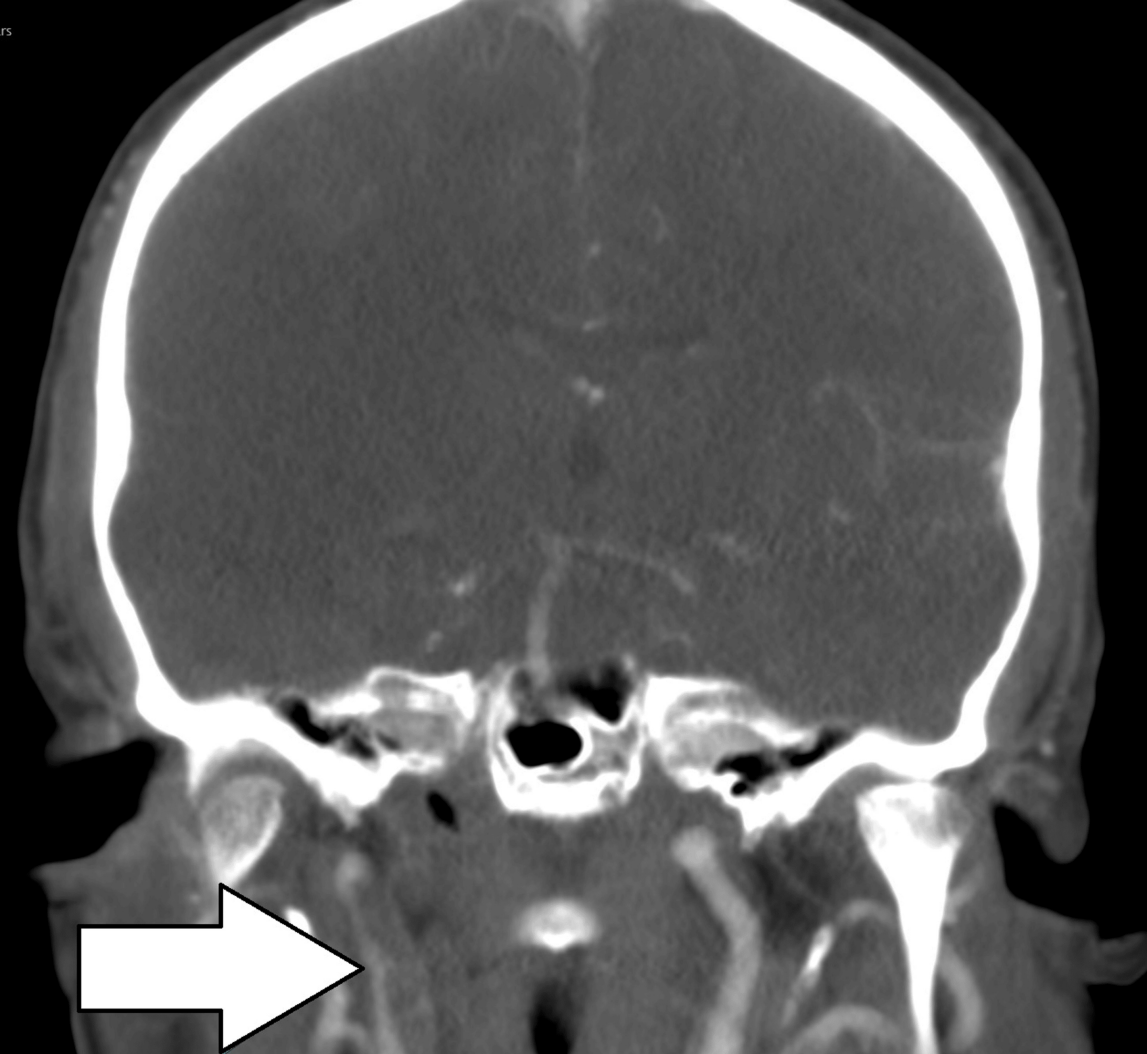
Repeat CT angiogram (coronal view) showing re-canalisation of the proximal right ICA (arrow). CT, Computed Tomography; ICA, Internal Carotid Artery

**Figure 7:**
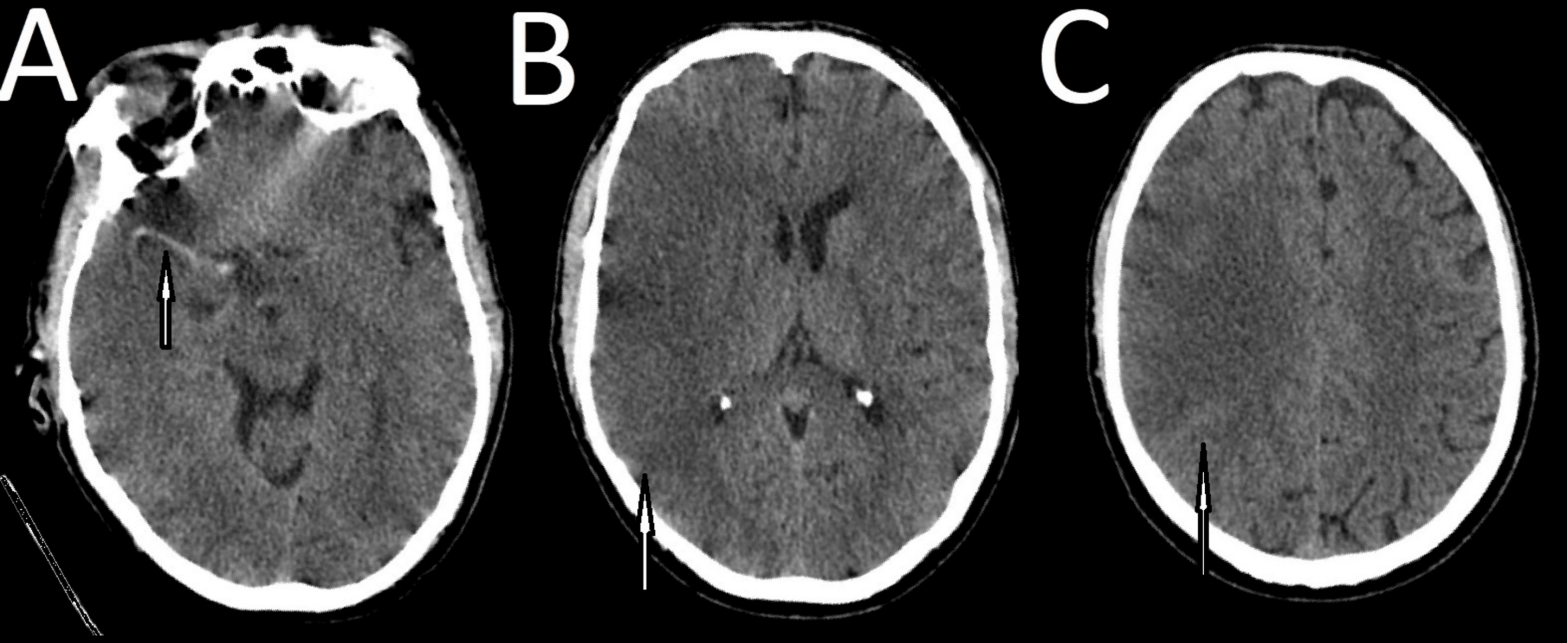
(A) Hyperdense MCA (arrow; the MCA subsequently clotted, with thromboses following the dissection); (B-C) established infarct with loss of grey-white matter differentiation (arrow; irreversible ischaemia), as well as cerebral swelling and mass effect (this is a relatively late sign of infarction and tends to be present several days following an infarct). MCA, Middle Cerebral Artery

A further repeat CT head and CT angiogram of the aortic arch and both carotids were performed to determine the progression of the dissection. These scans showed partial recanalisation of the right ICA in the previously occluded segment, with appearances compatible with dissection, and some evidence of flow reconstitution up to the level of the sella. However, in contrast to the previous day’s scan, there was very faint opacification of the supraclinoid ICA segment, as seen in Figures 6-7.

Following a cardiology review, the isoprenaline infusion was discontinued on the fifth day of admission, after continuous telemetry monitoring confirmed the absence of further bradycardic pauses. The patient remained on telemetry to monitor for any further cardiac events. An echocardiogram showed good left ventricular systolic function (ejection fraction, or EF > 55%), no evident thrombus, and no valvular pathology.

The patient continued to receive standard stroke care in the ward, including single antiplatelet therapy with aspirin for carotid artery dissection, along with intensive physiotherapy, occupational therapy, and speech and language therapy. He was also closely monitored, with regular neurological observations and vital signs assessment. Unfortunately, he continued to have cardiac pauses once the isoprenaline infusion was stopped. At this time, the pauses lasted approximately 26 seconds, and he remained normotensive. The isoprenaline infusion was restarted, with a rate variation of 8-10 mL/hour. We made multiple attempts to wean him off; however, these were unsuccessful and led to further episodes of symptomatic bradycardia. These episodes were characterised as syncopal bradycardias with recurrent sinus pauses, secondary to CSH. Other causes of syncopal bradycardia, including drug side effects, electrolyte imbalance, and transient neurological interference, were excluded.

On Day 6 of admission, the cardiology team decided that a permanent cardiac pacemaker was necessary to avoid further syncopal attacks and potential fatality, if the patient continued to experience syncope with bradycardia beyond a week, in line with European cardiology recommendations. The patient remained on the isoprenaline infusion until pacemaker insertion. On Day 9 of admission, he underwent implantation of a dual-chamber, rate-modulated permanent pacemaker (DDDR PPM). Immediately after PPM insertion, his symptoms resolved, and there was no further need for isoprenaline. He remained on telemetry throughout his inpatient stay.

The patient remained admitted for approximately three weeks. After two weeks of treatment with single antiplatelet therapy (aspirin), he was transitioned to clopidogrel 75 mg daily, in addition to ongoing symptomatic management for his post-stroke deficits. He received regular physiotherapy, which led to a notable improvement in motor strength. The stroke team was satisfied with his neurological recovery, and cardiology conducted multiple assessments following pacemaker implantation, expressing no further concerns. The patient remained medically stable throughout the remainder of his stay. He was ultimately discharged home once the occupational therapy team had arranged the necessary adaptive equipment and confirmed that he could safely manage daily activities with the support of his family.

## Discussion

This case underscores the intricate relationship between cerebrovascular accidents and underlying cardiovascular pathology, emphasising the need for a holistic diagnostic approach and multidisciplinary management. Prompt recognition of atypical presentations, and timely intervention, are paramount to optimising outcomes and mitigating long-term sequelae in such complex cases.

Ischaemic stroke due to ICA dissection is an uncommon but important cause of stroke in young men, with a peak occurring between 50 and 60 years of age. Associated risk factors include high blood pressure, oral contraceptive pills, smoking, and Marfan syndrome. Our patient did not have any of these factors, but his gender and age matched those in a few reported cases [9,10].

In ischaemic stroke secondary to carotid artery dissection, medical management remains the mainstay of treatment, as supported by current evidence and guidelines. The Cervical Artery Dissection in Stroke Study (CADISS), a randomised controlled trial, demonstrated no significant difference between antiplatelet and anticoagulant therapy in preventing recurrent stroke, thus supporting the use of either approach based on clinical context and individual risk factors [11]. Antiplatelet therapy, often with aspirin monotherapy, is generally preferred due to its favourable safety profile and ease of administration. There is no consensus to routinely recommend dual antiplatelet therapy (DAPT) in dissection-related stroke, except in specific situations, such as concurrent atherosclerotic disease or high embolic risk, where short-term DAPT may be considered. In our case, the patient was managed conservatively with aspirin alone, which is consistent with both the findings of the CADISS trial and local stroke treatment protocols.

CSH is seen with pressure at the site of the common carotid artery bifurcation, which produces a reflex that causes bradycardia and hypotension. CSH is defined as a ventricular pause of more than three seconds or hypotension of more than 50 mmHg. When the hypersensitivity does not respond in a usual manner to carotid sinus massage and causes syncope, it is referred to as CSS. Our patient fulfilled these criteria and thus was diagnosed with CSH. Formal carotid sinus massage was not performed due to the acute stroke setting. However, the diagnosis of CSS was made clinically, based on persistent symptomatic bradycardia with syncope and asystolic pauses documented on telemetry. We referenced the ESC 2018 guidelines, which support pacing for cardioinhibitory CSS when symptoms are recurrent and disabling, despite the absence of reversible causes. In one randomised controlled trial, the rate of syncope among non-paced CSS patients was 40%, as compared to 10% in patients who were paced [12].

CSH and CSS are commonly associated with ischaemic stroke, particularly in the setting of atherosclerotic carotid disease, where external compression or irritation of the baroreceptors may be more persistent due to fixed plaque burden. In contrast, carotid artery dissection is typically a transient condition, with gradual resolution as the arterial wall heals over time. However, in our patient, symptoms of CSS persisted beyond the typical timeframe seen with dissection-related cases, possibly due to sustained mechanical distortion or ongoing baroreceptor involvement in the acute phase. This highlights the need for individualised clinical assessment, as the pathophysiological impact of dissection may vary depending on its extent and location. While the ICA dissection was managed medically with antiplatelet therapy, the patient continued to experience symptomatic bradycardic episodes consistent with CSS. Given the persistence of these cardioinhibitory symptoms despite the stabilisation of the dissection and the absence of reversible causes, the decision was made to proceed with permanent pacemaker implantation for symptomatic relief and prevention of further bradycardia-related complications.

Importantly, in our patient, the dissection was located approximately 1.5 cm above the carotid bifurcation, slightly distal to the typical location of the carotid sinus, which is generally situated at the origin of the ICA near the carotid bifurcation [13]. Despite this, the patient exhibited classical signs of CSH and CSS. This suggests that even dissection just proximal or distal to the carotid sinus can cause mechanical distortion or lead to ischaemia of the baroreceptor-rich region, or its afferent pathways (e.g., glossopharyngeal and vagus nerves), which can trigger reflex bradycardia and syncope [6,7]. Such autonomic reflexes may be activated even when the dissection does not involve the exact anatomical site of the sinus, due to close proximity and neural network interconnections. Serial imaging and investigations did not point towards another cause of CSH and CSS. The pacemaker has been the preferred treatment for CSS, but few cases of dissection and aneurysm leading to permanent CSS have been reported. Additional data are needed for such cases [14].

While the exact cause of ICA dissection in our patient remains uncertain, possible contributing factors include spontaneous dissection, which is known to occur in otherwise healthy individuals without trauma, subtle mechanical stress, or intrinsic arterial wall weakness. Known risk factors for spontaneous dissection include connective tissue disorders, hypertension, recent infection, or minor neck movement or strain; however, none of these were clearly evident in our patient [15,16]. Persistent bradycardia in carotid artery dissection is not typically permanent; however, if it persists beyond one to two weeks, despite medical stabilisation and resolution of the acute phase, it may warrant permanent pacing - primarily when associated with cardioinhibitory syncope. ESC guidelines recommend pacing for cardioinhibitory CSS if symptoms are recurrent and disabling, especially after reversible triggers have been excluded [16]. However, we recognise that a direct causal relationship cannot be conclusively established in a single case, and we have explicitly stated that the findings and recommendations are based on an individual case report and should be interpreted with caution regarding broader clinical application.

## Conclusions

Our case highlights the diagnostic and therapeutic challenges posed by the convergence of ischaemic stroke and underlying vascular pathology. Comprehensive assessment, including neuroimaging and vascular studies, as well as multidisciplinary collaboration, are essential in guiding effective management strategies and optimising patient outcomes in intricate clinical scenarios. We recommend maintaining a low threshold for permanent pacemaker insertion in patients with carotid dissection who present with persistent cardioinhibitory symptoms, such as symptomatic bradycardia or sinus pauses. Although CSH and CSS are more classically associated with atherosclerotic disease at the carotid bifurcation, our case highlights that ICA dissection - without direct involvement of the bifurcation - can also provoke these reflexes, likely due to local mechanical distortion or ischaemia affecting baroreceptor pathways. Each case must be considered individually, with pacing decisions guided by symptom severity, duration, and failure to respond to conservative measures.
